# Facilitators and barriers of adaptation to diabetes: experiences of Iranian patients

**DOI:** 10.1186/2251-6581-13-17

**Published:** 2014-01-08

**Authors:** Hossein Karimi Moonaghi, Hossein Namdar Areshtanab, Leila Jouybari, Mohammad Arshadi Bostanabad, Heather McDonald

**Affiliations:** 1 Department of Post Graduate, Faculty of Nursing and Midwifery, Mashhad University of Medical Sciences, Mashhad, Iran; 2 Department of Medical - Surgical, Faculty of Nursing and Midwifery, Golestan University of Medical Sciences, Gorgan, Iran; 3 Department of Nursing, Faculty of Nursing and Midwifery, Tabriz University of Medical Sciences, Tabriz, Iran; 4 Health policy and quality officer at Seabird Island band, Chowat Rd, Agassiz, Vancouver, Canada

**Keywords:** Diabetes melitus, Psychological adaptation, Qualitative research

## Abstract

**Background:**

Diabetes mellitus is one of the most challenging and burdensome chronic diseases of the 21st century and More than 1% of the Iranian urban population older than 20 years develops Type 2 diabetes each year. Living with diabetes mellitus has been described as a dynamic personal transitional adaptation, based on restructuring of the illness perceived experience and management of the self. Adaptation to Type 2 Diabetes mellitus is an integral part of diabetes care.

This study explored the experiences of facilitators and barriers adaptation to Type 2 Diabetes by Iranian patients.

**Methods:**

This study was conducted by using qualitative content analysis. Data were collected via in-depth, semi-structured and face to face interviews with 15 patients with type2 diabetes.

**Results:**

Three themes emerged from collected data, including a) individual context with Beliefs, personal background, and previous experience subthemes. b) supportive system with Family, Society and Health organizations subthemes and c) self-comparison with comparison with other diabetes and comparison with other diseases subthemes.

**Conclusions:**

Identifying and managing Facilitators and Barriers adaptation to Type 2 Diabetes mellitus are an integral part of diabetes care. This study provides a better understanding of the factors from perspective of patients and it can be utilized by health care providers to adapt their health care and education contents to better meet the needs of people with diabetes.

## Introduction

Diabetes mellitus is one of the most challenging and burdensome chronic diseases of the 21^st^ century, and it is a growing threat to the world’s public health [1]. Diabetes mellitus currently affects about 285 million adults worldwide, and this figure is expected to rise to over 400 million adults by 2030 [2]. It is forecast that by 2030, 77.6% of diabetic patients will be from the developing countries [3]. Diabetes has increasingly become a great concern in the developing countries and Iran is no exception. Type 2 diabetes mellitus, a form of diabetes that is typically acquired in middle aged or older people, accounts for over 90% of all cases of diabetes [4]. The prevalence of type 2 diabetes is reported to be 1.3 to 14.5% in Iran [3]. The chronic nature of the disease and the severe complications result in pronounced lifestyle disruption, challenging psychosocial adjustment for the individual and substantial healthcare expenses [5].

Chronic illness acts as a stressor and initiates coping which can either contribute to or hinder adjustment [6]. Psychological, emotional, and social factors play important roles in chronic illness outcomes [7]. After the medical diagnosis of chronic illness [e.g., diabetes], patients are confronted with new situations that challenge their habitual coping strategies and go through a process of psychosocial adaptation [8]. The potential barriers to healthy coping are numerous. Some of them consist of low social support, financial stress or constraint, external locus of control, lack of access to providers and diabetes educators, low problem-solving ability [7].

Living with diabetes mellitus has been described as a dynamic personal transitional adaptation, based on restructuring of the illness perceived experience and management of the self. This means dealing with disabilities and limitations paired with a search for life meaning and realistic acceptable identities, that is, separating the person of the past from the person of the present, one that has to overcome personal and social adaptation process to diabetes [9]. Several studies investigated facilitators and barriers to management and adaptation to diabetes [10-15]. Cultural traditions and norms present in all populations, influence on practices of management and adaptation to disease [16].

The majority of studies are based on studies conducted in western societies that differ in terms of cultural and social backgrounds from the Eastern societies. Also the studies have shown that sociocultural content and belief systems could influence patients’ experiences in coping and adaptation to the illness [17,18]. The objective of our study is to explore the facilitators and barriers in adaptation to Type 2 diabetes mellitus among Iranian patients with using qualitative research methods. This methodology will allow us to gain deep knowledge about complex phenomenon Through exploring the experience and perceptions of those living with diabetes.

## Methods

The Ethics Committee of the Mashhad University of Medical Sciences approved this study. All participants were given oral and written information about the study, after which they consented to participate.

A qualitative study with a content analysis approach was used for the data collection and analysis. Qualitative research aims to explore, provide deep knowledge and understanding of the complex phenomenon under study that are encountered by clinicians, healthcare providers, policy-makers, and consumers in the healthcare system [19]. Qualitative content analysis focuses on the contextual meaning to “provide knowledge and understanding of the phenomenon under study [20].

Criteria for enhancing the rigor of qualitative studies have been proposed by Lincoln and Guba [1985] and include credibility, transferability, dependability and confirmability [21]. In the research process, researchers allocated sufficient time for data collection and have close communication with participants. The interviews returned the participants for verify the accuracy of results and validate the congruity of findings with their experiences. Member checking and ensuring that the researcher was represent their ideas. The data were coded and categorized independently by the authors and then emerged themes were compared. Opinion of experts and three PhD Candidate of nursing in data analysis as peer checking were used and discussed over a 2-week period. Regarding rigor, research team discussed and interpreted the findings until agreement was reached. These items enhanced the credibility of data. To increase the dependability, one of the researchers collected and analyzed the data and the other researchers checked and verified the results. Participants were selected by considering to maximum variation in terms of gender, age, previous experience of illness in family economic status, educational, job background and need or lack of need to injection of insulin to provide transferability.

### Data collection and analysis

#### *Participants*

Purposive samples of 15 patients were recruited. Patients were selected on the basis of the following inclusion criteria: willingness to participate in the study, a confirmed diagnosis of type 2 diabetes, passing at least 1 year after diagnosis of diabetes, awareness of their diagnosis and cognitively and physically able to participate in the study.

Data were collected via in-depth, semi-structured and face to face interviews conducted from September 2011 to August 2012. All interviews were private and conducted at the participant’s discretion with regard to place and time in Diabetes Association of Iran - Tabriz Branch.

The interviews were conducted in Persian by correspondence author. Each participant was interviewed only once .The analysis was conducted in Persian, and for the purpose of this article, translated into English. The interviewer asked the participants about their experiences of living with diabetes. The interviews were tape recorded and transcribed verbatim. The first interviews lasted 60–80 min and the next interviews lasted 40–50 min (mean time 45 min).

The analysis of collected data began once the first interview had been conducted and transcribed. Data were analyzed using qualitative content analysis techniques inspired by Graneheim and Lund man [22]. The analysis began by reading through each interview several times to obtain a sense of the whole of participants’ experiences. Then, the meaning units, which were words, sentences or paragraphs, that depicted important aspects of participants’ experiences of living with diabetes, were highlighted. These meaning units were then condensed to shorten statements that retain the content. In the next stage, these condensed meaning units, or codes, were abstracted from each interview transcript. Finally, using comparison, reflection and interpretation, these codes were grouped into categories and subcategories by the first three authors. Data collection continued until data saturation was reached. That is, data collection had gone on up to when no new code emerged from analysis of the data.

#### *Examples of interview questions are*

1. Can you tell about your experience of living with diabetes?

2. Can you identify some things you did or experienced that have helped you adapt to diabetes?

3. Can you identify some things you did or experienced those were not helpful in adaptation to your illness?

## Results

### Introducing the participants

Of the participants, 5[33.3%] were male and the average age was 47.8 ± 12.0 years, the mean duration of diabetes 11.7 ± 6.45, 10[83.33%] married ,8[53.3%] required insulin injection, 8[53.3%] low economic status and 5[33.3%] with previous experience of the illness in family. They had lower educational background (Illiterate to high school) and 6(40%) had higher educational background (college graduate) that females had a lower educational background than males and most were unemployed while most males were employed and three of them were retired.

During the data analysis, three main themes emerged as the participants’ experiences of facilitators and barriers in adaptation to the illness: Individual context, supportive system and self- comparison (Table 1).

**Table 1:** Themes and subthemes

***Theme***	***Subtheme***
**Individual context**	*● → beliefs about illness*
	*● → Personal background*
**Supportive system**	*● →* Family
	*● →* Society
	*● →* Human interactions in health organizations.
**Self- comparison**	*● → Comparison of diabetes mellitus with other diseases*
	*● → Comparison with other diabetes*

### Individual context

Individual context applied to all individuals involved in process of adaptation to diabetes. It refers to the individual’s background and resources that exist in persons. This category is comprised of three subcategories, which include: Beliefs, personal background, and previous experience.

#### *Negative beliefs versus positive beliefs about illness*

Some participants had negative beliefs about their illness. They believed that their illness is an incurable disease that ultimately results in the risk of death. Patients’ beliefs about health, illness, control and cure are predictive of the outcome of lifestyle. They tended to be the ones that also had trouble adapting/making the necessary lifestyle choices. Participants who had positive beliefs about the disease and treatment, as the disease can be controlled and were comfortable with it.

“*Having diabetes means death*.” *[woman 45 old]*

“Finally, when a person has been affected to diabetes, there is no cure for it and it is your life.” [man 56 old]

On the other hand, some participants had positive beliefs about their illness. They believed that their illness is a curable disease and may eventually be eradicated.

“*As were the days of dinosaurs and became depleted, doctors can cut diabetes generation. As they began to transplant pancreatic cells and the drug may be invented tomorrow and say you have a family history of this disease, take this medicine in order to prevent diabetes.” [man 50 old]*

#### *Personal background*

The study showed that adaptation to diabetes could be influenced by some demographic factors*: Economic status, Type of treatment [Require insulin injections or without insulin injections], life events and Previous experience of illness in the family.*

• *Economic status can be affected on adaptation to diabetes.* Low economic status creates difficulties in providing medical facilities and services. On the other hand, high economic status is a facilitator to adapt to diabetes. Because it can easily meet the therapeutic needs of diabetics patients.

“*I am eating medication for my diabetes. They are expensive and sometimes difficult to get them prepared.” [woman 56 old]*

• *Type of treatment received by participants can be affected on adaptation to diabetes*. Most participants perceived insulin therapy as the most bothersome and less feasible treatment compared with oral hypoglycemic agents.

“Sometimes I forget to inject insulin on time. After the injection, you forget to eat food and drop blood sugar, or you are a guest and you did not find time to do insulin injection on time.” [woman 46 old]

• *Stressful life events may influence metabolic control indirectly by detracting from self -care behavior*. On the other hand, as facilitator, pleasurable life events promote the motivation, energy, performance and coping abilities.

“My wife death and conflict about heritage between my children bothered me. As a result, my blood sugar went up.” [woman 60 old]

On the other hand, the pleasant life events act as a facilitator in adaptation to diabetes.

“After getting married and reintegrate children, I feel a little relieved, and sometimes I play with the grandchildren.” [man 55 old]

“After the birth of my kids I feel like I have great enthusiasm for living with the disease.” [woman 60 old]

• *Prior experience with a family member with diabetes can be affected on adaptation to diabetes.*experienced participants were sensitive to their health and preventive actions were done.

“Because both my father and my mother was diabetic and had a hereditary issue every six months or once a year I go to the doctor… to control.” [woman 54 old]

### Supportive system

Supportive system applied to all individuals involved in process of adaptation to diabetes. It refers to environmental background and resources that exist for persons. This category is comprised of three subcategories, which include: Family, Society and Human Interactions in Health Organizations.

#### *Family*

Participants’ statements indicated that family support was a critical aspect of adaptation to diabetes. In particular, participants discussed receiving support from their partners. It primarily served as a facilitator in help with diet and living in harmony. On the other hand, sometimes family members do not understand the patient’s condition. It acts as barrier in adaptation to diabetes.

“My wife gives me comfort. She advised me to eat vegetables scheduled to attend the program in any way I eat vegetables and salads.” [man 61 old]

“I live alone and I live apart from my children.”[woman 55 old]

#### *Community*

Individual ability in adaptation to diabetes cannot be separated from community context and support for diabetes care. Some researchers emphasize social resources as the “fundamental causes” of health disparities. These factors both shape health behaviors and influence health directly. Many participants commented on the role of media as an important source of information, general Sport and leisure facilities and health insurance.

• *Providing of confusing or conflicting health Information versus Providing clear and explicit health Information:* Health messages on television and other mass media have the potential to significantly influence the public’s health-related knowledge and behaviors. Unstable and ambiguous health messages published media causes confusion and conflict in understanding the disease and living with it.

“One says do not eat, other says eat, there is no medical stability. Check to see what is good for us” [man 58 old] [Confusing health information].

• *General sport and leisure facilities versus lack of general Sport and leisure facilities:* Public sports and recreation facilities, easy access to them and leisure activities help to cope with stress, stimulate positive emotions and reduce or buffer negative ones.

*“Public parks must be equipped sports hall, Free sports hall must be available to be able to use them, especially in winter.” [man 45 old] [*Lack of access to recreational facilities]

• *Inefficient health insurance versus health insurance:* Health insurance can play an important role in access to medical care services, and people who do not have access to health insurance have less possibility of accessing health care services.

“*Some drugs are not covered by insurance and I’m prepared to pay a high cost.” [woman 50 old]*

#### *Human interactions in health organizations*

Participants’ statements indicated that health organizations were a critical aspect of adaptation to diabetes. More participants emphasized on the human interactions and how to provide health information in these organizations. The lack of a common language between doctor and patient, Lack of understanding of the patient’s condition by the doctor and the lack of clear direction for advice about some of the privacy problems [e.g., sexual problems] can lead to continuing problems in living with the disease.

“I’m very sad that my previous doctor was not. Because current doctor talking in Farsi and I did not know the language, I do not understand something.” [woman 65 old]

“Following this illness, sexual problems arise, there is no one that could clear guidance in this regard and help you in this case.” [man 56 old]

On the other hand, the use of plain, understandable language with respect to patient by physician and Refer the patient to appropriate resources were as facilitator in adaptation to diabetes.

“Doctor treated me very politely and humanely and to listen to my words. Then he spoke about my disease and its treatment in clear and understandable language to me.” [man 58 old]

“After my discharge from the hospital and introduced me to the Diabetes Association. give me books about diabetes education and I read them, and I saw a person with diabetes can live.” [man 51 old]

### Self-comparison

Patients living with the disease over the years, their disease and their health conditions are compared with other diseases and the other diabetes. Then image of the disease in their mind is made up. This may be positive or negative. As a result, it can act as a facilitator or a barrier in patient adaptation to the disease.

Self-comparison applied to all individuals involved in process of adaptation to diabetes. This category is comprised of two subcategories, which include Subjective image of their disease compared with other diseases and Subjective image of their condition compared with other diabetes. Many participants commented on Positive image of their disease and focus on the positive aspects of disease.

#### *Comparison of diabetes mellitus with other diseases*

Many participants believe that compared with other diseases, diabetes is light and small one.

“Diabetes is a disease which is in contrast to much of diseases is shahaneh.”

What do you mean shahaneh? [interviewer].

“That in comparison with hepatitis or cancer is mild.” [man 40 old]

However, some participants believe that, diabetes compared with other diseases is bad.

“Diabetes compared with other diseases for example cancer is good. Because the cancer may make the breast a lift or chemotherapy. But compared with conditions such as bone fractures, heart disease is bad.” [woman 50 old]

#### *Comparison with other diabetes*

Many participants believe that their condition compared with other diabetes is better.

“With this condition, I thank God that I have eyes and I can see, I have foot and I can walk .Because there are diabetes who have lost their vision due to disease or feet are amputated or have had kidney problems.” [woman 50 old]

On the other hand, some participants believed that their condition compared with other diabetes is worse.

“when I meet other diabetes in park or party , I feel that with this disease have difficulty seeing over time, and I'm wearing glasses. One of my toes was amputated and cannot walk properly. I have broken.” [man 65 old]

## Discussion

In this study, the factors mentioned in individual context as facilitators and barriers can be involved in emotional and cognitive responses to adaptation to diabetes. This demonstrates the need for interventions to modify some of the factors [e.g., the improved income, recognition and effective factors in adding insulin to oral hypoglycemic therapy, modify negative beliefs and remember and focus on the pleasurable life events]. The perception and belief that illness is curable/controllable is positively related to psychological well-being, social functioning and vitality, but negatively related to psychological distress and the disease state [23]. As barrier, Psychological resistance to insulin therapy can result from a range of personal viewpoints involving cognitive appraisal and emotional reactions in adults with type 2 diabetes [24]. Distressful life events may influence metabolic control indirectly by detracting from self -care behavior. Stressful life events might increase psychological distress which has these metabolic effects [25,26]. On the other hand, as facilitator, pleasurable life events promote the motivation and energy, performance and coping abilities. Prior experience with a family member with diabetes could result to susceptibility to your health. Once a person perceived himself as being susceptible to diabetes, he/she forms intentions to take preventive actions. In the study, women participants were sensitive to their health and actively participated in follow-up examinations. Women are more likely than men to seek medical help for chronic illness [27]. Thus, taking into account existing studies, it seems that individual context is as facilitators and barriers in management and adaptation to diabetes [28-32].

In supportive system as facilitators and barriers factors such as family members support, general Sport and leisure facilities, good interaction between patient and doctor and Provision of clear and consistent information by Media and health staff were as facilitators adaptation to diabetes. On the other hand, lack of support from family members, provide Inconsistent and unclear information by Media and health staff, poor interaction between patient and doctor and Inefficient health insurances were as barriers adaptation to diabetes. Conflicting health information could leave even highly educated people confused about how to adopt appropriate heath behaviors in order to maintain good health. In addition, the confusing health information can result in suspicion about message credibility, and perceived unreliability of the health campaign [33]. Health insurance is a significant predictor of access to medical care services, and people who do not have access to health insurance have less possibilities of accessing health care services [34].

The factors mentioned in supportive environment (family, community, health organizations) can be involved in reorganize, and restructure the individual context. That was outside the scope of the patient. Therefore, it seems that these factors are as facilitators and barriers in management and adaptation to diabetes [28-33,35-39].

In self- comparison, most participants had Positive image of their disease and focus on the positive aspects of disease and diabetes compared with other diseases is light and minor one. It can enhance subjective wellbeing and self-enhancement. Studies have showed that Positive comparisons are used by people who feel stressed or threatened to enhance positive affect and self-image. Studies have showed that Positive comparisons are used by people who feel stressed or threatened to enhance positive affect and self-image. It is associated with better adjustment to illness It is associated with better adjustment to illness [40]. Hence, it seems that these factors are as facilitators and barriers in management and adaptation to diabetes [41,42].

In summary, Self-comparison is usually caused by the interaction of the individual context and supportive system. This means that suitable individual context with effective support systems can lead to positive self-comparison. Psychosocial adaptation is a process involving internal and external factors. Persons with type 2 diabetes mellitus, who adapt well, accept the reality of the disease, reorganize, and restructure the environment so they will have a meaningful and purposeful life with the disease [43].

## Conclusion

This study contributes to our understanding Facilitators and barriers of adaptation to disease among diabetes. The results of this study can be utilized by health care providers to adapt their health care and education contents to better meet the needs of people with type 2 diabetes mellitus. These results suggest that clinicians need to stimulate the enabling facilitators, remove barriers to adaptation and strive to develop a better understanding of the contextual factors influencing it. In education flied can utilize in education of nursing students and physicians and families of diabetes. The results of this study can utilize in next researches for developing appropriate tool based our cultural context to study effective factors in adaptation to diabetes in large populations.

### Limitations of the study

In this study, samples were selected only from urban communities. Also, findings reflect only the view of patient regarding facilitators and barriers adaptation to type 2 diabetes. Thus, as with other such qualitative studies, caution is needed in generalizing the findings to either Iran as a country or other countries around the world. Also future study requires input from both health care providers and families. We believe that it is necessary to conduct a population-based survey to confirm the finding.

## Competing interests

The authors declare that they have no competing interest.

## Authors’ contributions

All authors approve the content of the manuscript and NAH was the principal researcher and performed the interviews. KMH and JL performed the qualitative analysis. NAH and ABM wrote the first draft of paper, MH contributed to editing the manuscript and all authors read and approved the final manuscript.
